# A large left ventricular pseudoaneurysm in Behçet's disease: a case report

**DOI:** 10.1186/1471-2482-5-13

**Published:** 2005-06-14

**Authors:** Seyed Mojtaba Marashi, Payam Eghtesadi-Araghi, Mohammad Hussein Mandegar

**Affiliations:** 1 Assistant Professor of Anesthesiology, Department of Cardiac Surgery, Dr. Shariati Hospital Complex, Tehran Univ. of Medical Sciences, Tehran 14197 Iran; 2 Anesthesiologist, President of Parsteb Pajouheshyar Medical Sciences Research Institute (NGO), Department No. 5, 37^th ^(Eastern), First Golzar St., Ashrafi Esfahani Bulv., Ponak Sq., Tehran 1476783476, Iran; 3 Associate Professor of Cardiac Surgery, Department of Cardiac Surgery, Dr. Shariati Hospital Complex, Tehran Univ. of Medical Sciences, Tehran 14197 Iran

**Keywords:** Behçet's disease, cardiac, left ventricular, pseudoaneurysm

## Abstract

**Background:**

Behçet's disease is a collagen-vascular disease most commonly seen in Asia and Mediterranean area. Different organs and systems including cardiovascular system could be involved. Pseudoaneurysm is the most common form of arterial involvement in Behçet's disease; however, cardiac pseudoaneurysm is rare.

**Case Presentation:**

A rare case of 13 years old boy with a 4-year history of Behçet's disease with development of a huge left ventricular pseudoaneurysm is reported who had been admitted because of cough, chills, fever, and chest pain. Findings obtained on echocardiography, magnetic resonance imaging, chest computed tomography and coronary angiography confirmed a left ventricular pseudoaneurysm. There was no complication for next 24 months follow up period after surgical treatment.

**Conclusion:**

Considering its fatality and nonspecific manifestations, one should consider cardiac pseudoaneurysms as a potential risk in any patient with Behçet's disease.

## Background

In 1937, Hulusi Behçet, a Turkish dermatologist, first described a chronic autoimmune disease bearing his name with characteristic orogenital aphtous ulceration and uveitis [1]. The etiology of Behçet's disease, which is most commonly seen in Asia and Mediterranean area, is still unknown. The primary pathology is a vasculitis affecting skin, joints, pulmonary, gastrointestinal, urinary, and nervous systems [2]. Its vascular complications are most frequently manifested as thromboembolism in veins and pseudoaneurysm in arteries [3]. Although pseudoaneurysms are the most common form of arterial involvement in Behçet's disease, we could only find one case reported by Rolland *et al. *[4] with Behçet's disease and cardiac pseudoaneurysm. Occasional cases of cardiac pseudoaneurysms have been reported in association with rheumatoid arthritis [5] and Kawasaki's disease [6]; however large cardiac pseudoaneurysms are mostly complications of cardiac surgery, myocardial infarction, endocarditis, and chest trauma [7].

In this report we present a patient with Behçet's disease and a huge left ventricular pseudoaneurysm.

## Case presentation

A 13 years old boy with Behçet's disease was referred to our hospital with chills, fever, cough, and chest pain of one month duration in June 2001. The diagnosis of Behçet's disease was established 4 years prior to this admission, that had presented with oral aphtae, orchitis, right eye uveitis leading to blindness, recurrent pseudofolliculitis, knee arthritis, and lower extremity deep vein thrombosis, all attributable to this autoimmune disorder. He had been treated by Prednisolone (15 mg/day) and Methotrexate (7.5 mg/week) for last 7 month.

**Figure 1:**
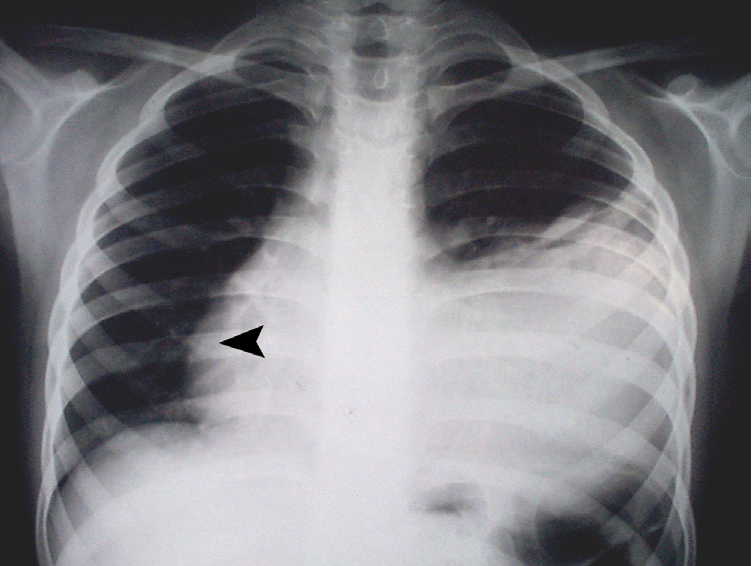
The PA chest X-ray: left-sided pleural effusion and a large mass in anterolateral part of left lung which had overshadowed the left border of the heart. Arrowhead indicates shift of the heart to the right side.

**Figure 2:**
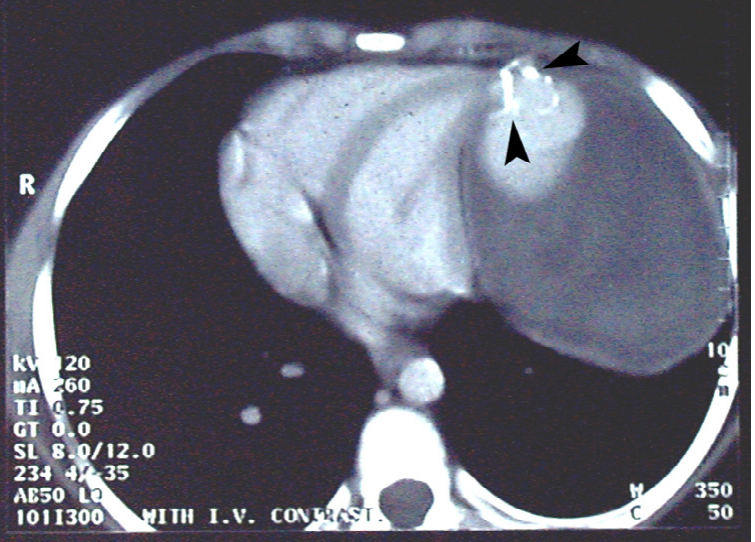
Chest computed tomography scan with contrast at the level of T7 showing the large pseudoaneurysm. The lesion was a well-defined partially calcified mass with tubular density adjacent to the heart. Arrowheads indicate the calcifications. It was filled with contrast medium concurrently with the heart. This lesion, which was mostly occupied by thrombosis, had a mass effect on heart.

**Figure 3:**
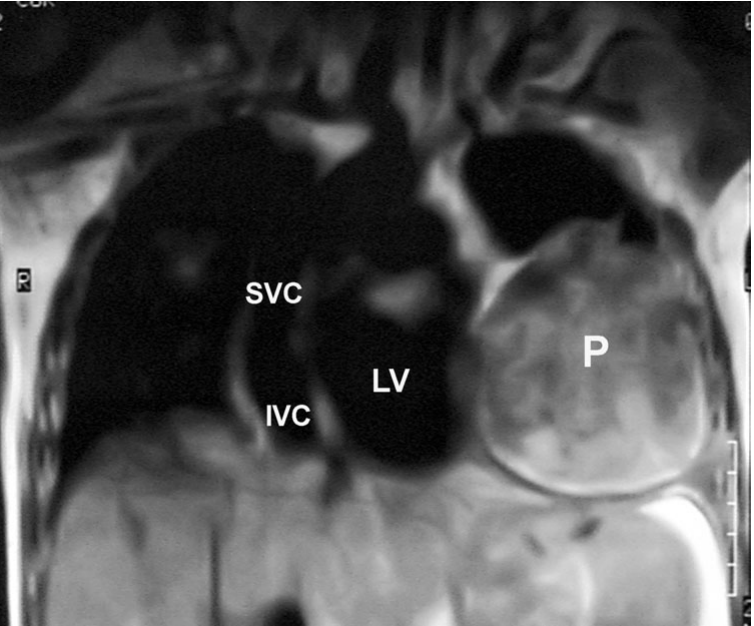
The MRI indicated a mass with inhomogeneous signals implying the presence of blood and clots in addition to calcification. (SVC = Superior Vena Cava; IVC = Inferior Vena Cava; LV = Left Ventricle; P = Pseudoaneurysm)

Physical examination revealed III/VI to-and-fro murmur along the left sternal border and an S3 gallop. The posterior-anterior chest X-ray (Fig. 1), computed tomography scan with intravenous contrast media at the level of T5–T8 (Fig. 2), magnetic resonance imaging (MRI) (Fig. 3), coronary angiography and echocardiography all revealed a 10.1 × 14.8 cm left ventricular pseudoaneurysm in the anterior wall of left ventricle. There was no narrowing suggesting coronary artery disease in his coronary angiography. In two-dimensional echocardiography, the typical features of pseudoaneurysm was noted including a relatively narrow neck in comparison with the diameter of the aneurysm, sharp discontinuity of the endocardium at the site at which the aneurysm communicates with the left ventricle, no noticeable valvular dysfunction, and left ventricular wall motion abnormalities. The orifice to radius ratio was not measurable due to the large size of pseudoaneurysm.

**Figure 4:**
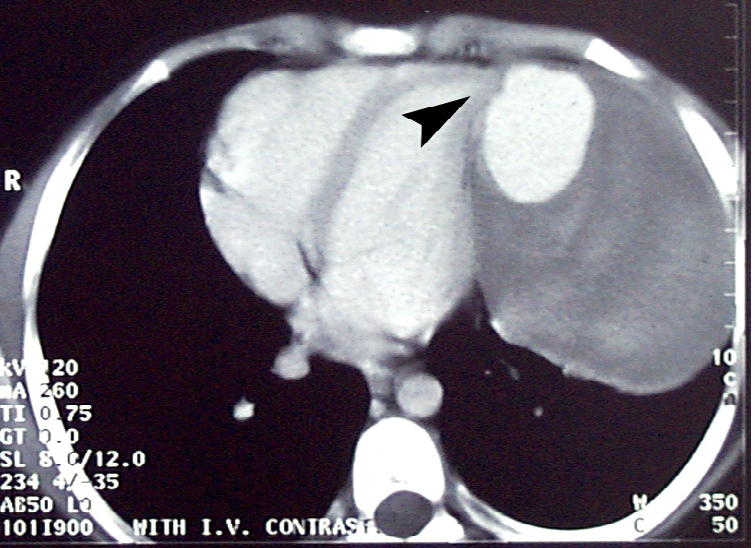
Chest computed tomography scan with contrast at T7 level. Arrowhead marks the ostium.

At surgery, the ostium (arrowhead in Fig. 4) was measured 2.5 × 3.0 cm. The above mentioned results warranted a surgery with median sternotomy approach. After pericardotomy, a pulsatile mass appeared at the tip of left ventricle with a fistula to the heart. The pseudoaneurysm and large amounts of thrombus within it were resected, and the defect in the left ventricular wall was repaired by Teflon Plegeted Prolen 4/0. The patient made an uneventful recovery. The pathologic examination revealed a fibrous pseudoaneurysm including areas of old hemorrhage and thrombosis and chronic inflammation. There was no complication for next 24 months follow up period after the operation.

## Conclusion

We have presented an unusual patient with Behçet's disease and a large (10.1 × 14.8 cm) left ventricular pseudoaneurysm. Behçet's disease is a systemic disorder with mucocutaneous, ophthalmic, neurological, cardiovascular, pulmonary, gastrointestinal, urogenital and musculoskeletal involvement. Its vascular manifestations are thrombophlebitis and, less frequently, arterial lesions such as pseudoaneurysms, occlusions and stenoses [8]. About 8% of the patients with Behçet's disease have severe vascular complications such as arterial pseudoaneurysms and occlusions [1]. Pseudoaneurysms are the most common form of arterial involvement in Behçet's disease [3].

Cardiac involvement is rare in Behçet's disease [9] and engages only about 6% of patients [4]. Cardiac involvement in this disorder is a diffuse process that involves both cardiac structure and vascular elements. Higher incidences of interatrial septum aneurysm (6% to 31%), mitral valve prolapse (3% to 25%), mitral regurgitation (6% to 40%), and aneurysmal dilatations of valsalva sinus and ascending aorta were observed in the Behçet's disease patients than in the normal subjects [10]. Pericarditis, myocarditis, endocardial fibrosis, conduction defects, and aortic regurgitation were also observed [11]. Although left ventricular aneurysms with Behçet's disease have occasionally been reported, we found only one case of cardiac pseudoaneurysm in these patients reported in literature. Rolland *et al. *[4] reported a 29 years old patient with Behçet's syndrome and a false left ventricular aneurysm and coronary artery aneurysm, which were repaired under cardiopulmonary bypass with no postoperative complications.

Cardiac pseudoaneurysm is defined as a rupture of the myocardium that is contained by pericardial adhesions or the epicardial wall. This phenomenon can be explained by myocardial fragility induced by ischemia due to vasculitis process of Behçet's disease [4]. Because there was no evidence suggesting coronary artery disease in his coronary angiography of our patient, it could be assumed that this pseudoaneurysm was resulted from rupture of the left ventricle due to angiitis and the myocardial fragility induced by ischemia.

In contrast to a true ventricular aneurysm, in which the wall is composed of myocardial scar tissue, the wall of a pseudoaneurysm is composed of thick fibrous tissue and pericardium [12]. In our case, pseudoaneurysm was consisted of profuse fibrous tissues and thromboses in various stages.

Cardiac pseudoaneurysms have the potential to leak or rupture and can be the source of peripheral emboli [10]. Different reports have discussed that such a contained rupture has a greater propensity for rupture than a true aneurysm, whose wall contains myocardium. Rupture of a left ventricular pseudoaneurysm is usually fatal; hence appropriate recognition and treatment (early surgery) even for asymptomatic patients is strongly recommended [7].

The diagnosis of pseudoaneurysm is not straightforward and is rarely suggested by clinical signs and symptoms [7]. In our patient the pseudoaneurysm presented with nonspecific symptoms and signs. Thus, such a diagnosis was highly unlikely before getting the results of imaging. Various imaging methods have been used to diagnose pseudoaneurysm, including two-dimensional and contrast echocardiography [13], computed tomography, magnetic resonance imaging, and left ventricular angiography. Each has its advantages and disadvantages but echocardiography has become the most common examination used for first diagnosis because it can evaluate other associations such as valvular regurgitation, thrombus formation, and ventricular function, are often important supplements to clinical management [7].

Like our patient, chest radiography sometimes shows a localized bulge on the cardiac silhouette. On computed tomography, pseudoaneurysms are characterized by an abrupt disappearance of the myocardial wall at the border of the pseudoaneurysm. Magnetic resonance imaging shows the low signal of the pericardium, which constitutes the only wall of the pseudoaneurysm [7].

Surgical repair is usually recommended when a left ventricular pseudoaneurysm is detected [14,15]. In this case surgical intervention was mandatory, partly due to the young age of the patient. However, in cases of post-infarction left ventricular pseudoaneurysm, which is one of the most common causes, routine surgical repair regardless of other clinical characteristics of the patient remains as a matter of discussion. Some authors believe that the necessity of surgical repair in these cases should be individualized for each patient [7,16].

In patients with post-infarction left ventricular pseudoaneurysm, surgical repair of pseudoaneurysm was associated with an acceptable surgical mortality rate [7] and the long term outcome appears relatively benign [13] and late death was related primarily to the underlying disease or cardiac dysfunction [7]. There is no data on the long term prognosis of patients with cardiac pseudoaneurysms in Behçet's disease; however, long-term survival could not be expected given the diffuse involvement of cardiac structure and vascular elements [10].

Considering its fatality and nonspecific manifestations, one should consider cardiac pseudoaneurysms as a potential risk in any patient with Behçet's disease. Thanks to early diagnosis and surgery, our patient was treated successfully and had no complications in a follow-up period of 24 months.

## Abbreviations

SVC = Superior Vena Cava

IVC = Inferior Vena Cava

LV = Left Ventricle

## Competing interests

The author(s) declare that they have no competing interests.

## Authors' contributions

SMM: Data collection, participated in the design of the study, critical review of the manuscript.

PE: Conceived the study and wrote the manuscript, participated in the design of the study.

MHM: Supervisor and conductor of treatment team and critical reviewer of the manuscript.

All authors read and approved the final manuscript.

## Pre-publication history

The pre-publication history for this paper can be accessed here:


